# Tumor-derived extracellular vesicles for the active targeting and effective treatment of colorectal tumors *in vivo*

**DOI:** 10.1080/10717544.2022.2105444

**Published:** 2022-08-08

**Authors:** Van Du Nguyen, Ho Yong Kim, You Hee Choi, Jong-Oh Park, Eunpyo Choi

**Affiliations:** a School of Mechanical Engineering, Chonnam National University, Buk-gu, Gwangju, Korea; b Korea Institute of Medical Microrobotics, Buk-gu, Gwangju, Korea

**Keywords:** Doxorubicin, extracellular vesicle, chemotherapy, colorectal cancer, nanoparticle drug delivery

## Abstract

Colorectal cancer remains one of the main causes of cancer-related deaths worldwide. Although numerous nanomedicine formulations have been developed to tackle the disease, their low selectivity still limits effective therapeutic outcomes. In this study, we isolated extracellular vesicles (EVs) from CT26 colorectal cancer cells and 4T1 murine mammary carcinoma cells, loaded them with the chemotherapeutic agent (doxorubicin, DOX). Then we evaluated the cellular uptake of the extracellular vesicles both in 2D monolayer and 3D tumor spheroid setups using confocal laser scanning microscope and flow cytometry. *In vivo* tumor homing of the extracellular vesicles was verified on CT26 tumor bearing BALB/c mice using *in vivo* imaging system. Finally, *in vivo* therapeutic effects were evaluated and compared using the same animal models treated with five doses of EV formulations. CT26-EV-DOX exhibited excellent biocompatibility, a high drug-loading capacity, controlled drug release behavior, and a high capability for targeting colorectal cancer cells. In particular, we verified that CT26-EV-DOX could preferentially be up taken by their parent cells and could effectively target and penetrate 3D tumor spheroids resembling colorectal tumors *in vivo* in comparison with their 4T1 derived EV partner. Additionally, treatment of colorectal tumor-bearing BALB/c mice with of CT26-EV-DOX significantly inhibited the growth of the tumors during the treatment course. The developed CT26-EV-DOX nanoparticles may present a novel and effective strategy for the treatment of colorectal cancer.

## Introduction

Colorectal cancer is one of the main causes of cancer-related deaths worldwide (Sung et al., 2021). At present, surgical removal of the tumor mass remains the primary treatment method for this disease. However, the rates of recurrence and metastasis remain relatively high, at approximately 50% of patients (Gu et al., 2019). Although chemotherapy is employed as an alternative treatment option, the severe side effects caused by the drugs and the eventual resistance of the tumor cells to the chemical agents still lead to poor therapeutic outcomes.

Recently, targeted drug delivery via nanoparticles has emerged as a promising method for enhancing the efficacy of chemotherapeutic agents. Using this approach, the loaded drugs are also protected within the nanoparticles, with liposomes being widely employed for this purpose. Liposomes are nanoparticles composed of a lipid bilayer that covers an aqueous core, thereby facilitating both hydrophobic and hydrophilic drug encapsulation (Nguyen et al., 2020). Recently, two nanoliposomal formulations encapsulating oxaliplatin (Sankhala et al., 2009) and irinotecan (Chibaudel et al., 2016), respectively, were developed and their effectiveness against colorectal cancers was studied in clinical trials. Unfortunately, these formulations did not show selectivity for colorectal cancer cells (Gu et al., 2019).

Extracellular vesicles (EVs), which have similar structures to liposomes, are organelles that are secreted to the extracellular environment by numerous types of cells. They offer several advantages over liposomes and other synthetic nanoparticles, such as prolonged stability in body fluids, an extended half-life, and decreased live location after systemic administration (Yu et al., 2018). Additionally, since EVs can be derived from the patient’s own cells, they would have excellent biocompatibility and may be less immunogenic when being injected back into the same patient after engineering (Tian et al., 2014). Moreover, the different surface proteins (e.g. integrin, tetraspanins, and other adhesion proteins) expressed on EVs would promote their uptake by cancer cells (Zhang et al., 2018).

Since their discovery, EVs have been isolated from a variety of cell types, such as stem cells (Kim et al., 2018; Hosseini Shamili et al., 2019; Mathew et al., 2019; Kim et al., 2020), natural killer cells (Zhu et al., 2017, 2019), macrophages (Jia et al., 2018; Kim et al., 2018; Wang et al., 2018) (Table S1), and especially tumor cells (Qiu et al., 2019; Yong et al., 2019; Qiao et al., 2020; Xie et al., 2021) (Table S2) and applied for drug/gene delivery purposes to treat different types of cancer. However, the development of an EV formulation for effective colorectal cancer treatment has still not been achieved.

**Figure 1.**
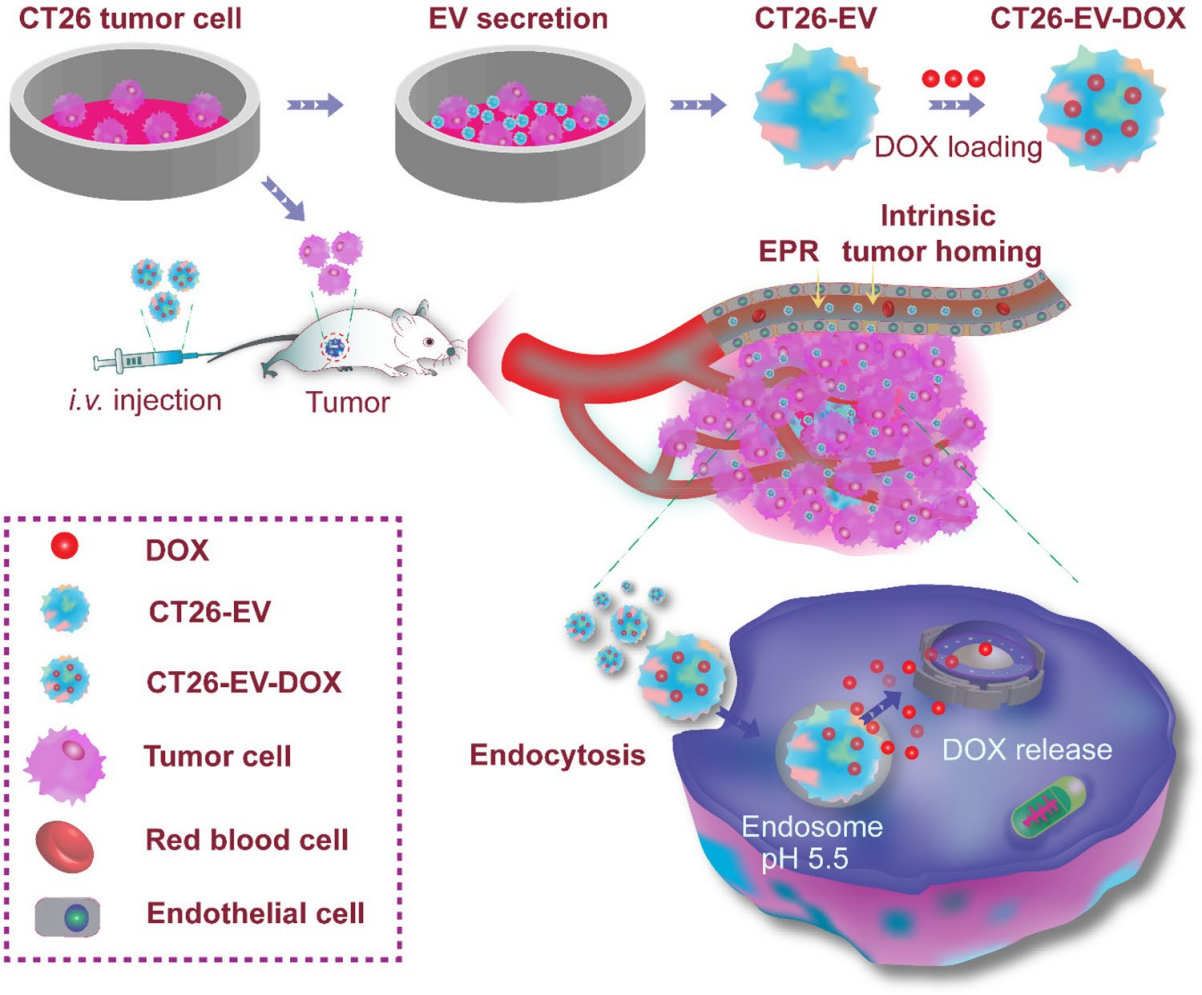
Schematic diagram illustrating the isolation of colorectal tumor cell-derived extracellular vesicles and their engineering into drug-loaded nanoparticles (CT26-EV-DOX) for the targeting and treatment of colorectal tumors. DOX, doxorubicin; EV, extracellular vesicles; CT26, murine colorectal cancer cells.

Therefore, in this study, we isolated EVs from the CT26 murine colorectal cancer cell line, loaded them with the anticancer drug doxorubicin (DOX), and then assessed the performance of the engineered CT26-EV-DOX nanoparticles both *in vitro* and *in vivo* (Figure 1). We showed that CT26-EV-DOX possessed excellent biocompatibility, high drug-loading capacity, controlled drug release behavior, and high capability for targeting colorectal cancer cells. In particular, we confirmed that the drug-loaded EVs could effectively target and penetrate 3D tumor spheroids resembling colorectal tumor cells under *in vivo* conditions. Furthermore, *in vivo* experiments on colorectal tumor-bearing BALB/c mice showed that five injection doses of the drug-loaded EVs could significantly inhibit the growth of the tumors. As such, our novel CT26-EV-DOX nanoparticles may be an effective alternative nanomedicine for the treatment of colorectal cancer.

## Materials and methods

### Extracellular vesicle isolation and analysis

First, 5 × 10^6^ CT26 cells (Korean Cell Line Bank, Seoul, Korea) were cultured in RPMI-1640 medium supplemented with 10% EV-depleted fetal bovine serum and 1% penicillin and streptomycin in 10-cm cell culture plates. The conditioned media were harvested after 48 h for isolation of the CT26-EVs using a previously described differential ultracentrifugation method (Yong et al., 2019) with minor modifications. In brief, 10 mL of the medium was sequentially centrifuged at 1000 ×*g* for 10 min and at 10,000 ×*g* for 30 min to remove cell debris and dead cells. The supernatant was retained and centrifuged for 90 min at 100,000 ×*g* and 4 °C in an ultracentrifuge (CP100NX Himac, Koki Holdings, Tokyo, Japan), following which the EV-containing pellet was suspended in phosphate-buffered saline (PBS) for analysis. After lysing the EVs with radioimmunoprecipitation (RIPA) buffer (P8100, GenDEPOT, Barker, TX, USA), their protein concentration was measured using the bicinchoninic acid (BCA) protein assay. First, 25 μL of the samples and protein standards were, respectively, dispensed into the wells of a 96-well plate, and then 200 μL of BCA reagent (reagent A/reagent B = 50/1, v/v) was added to each well with thorough mixing for 30 s. After 30 min of incubation at 37 °C in the dark, the absorbance of the mixture in each well was measured at the wavelength of 562 nm using a microplate reader. The protein concentration was determined on the basis of the standard curve (Zhang et al., 2018). For performance comparisons, we applied the same methods to prepare 4T1-EV-DOX nanoparticles using EVs from 4T1 murine mammary carcinoma cells (American Type Culture Collection, Manassas, VA, USA). A scheme of the processes used to engineer the DOX-loaded EVs is presented in Figure 1.

### Transmission electron microscopy imaging of the extracellular vesicles

To observe the various EVs by transmission electron microscopy (TEM) imaging, 5 μL of the engineered nanoparticles suspended in deionized water was dispensed onto a carbon-coated copper TEM grid and air-dried for 15 min at ambient temperature. The EVs were then examined under a TEM system. Additionally, the various EVs in PBS were passed through a 0.45 μm filter twice, and their average size was then determined by dynamic light scattering using a Zetasizer Nano analyzer (Qi et al., 2016; Zhang et al., 2017).

### Western blot assay of extracellular vesicle markers

A RIPA buffer containing a protease inhibitor (1×) was used to lyse the EV pellets (harvested as described above) at 95 °C for 5 min. Then, equal amounts of protein (20 μg) were resolved on a 10% sodium dodecyl sulfate-polyacrylamide gel, following which the protein bands were electrotransferred to a polyvinylidene difluoride membrane at 4 °C using a voltage of 100 V, a constant current of 0.1 A, and a run time of 1 h 20 min. Next, the membrane was incubated with 3% skim milk in Tween-containing Tris-buffered saline (TBS-T: 10 mM Tris, pH 8.0, 150 mM, and NaCl solution containing 0.05% Tween 20). Then, after three washes with TBS-T (10 min each), the membrane was incubated overnight at 4 °C with primary antibodies (diluted 1:1000 in 3% skim milk) targeting the following proteins: CD63 (ab217345, Abcam, Cambridge, UK), tumor susceptibility gene 101 protein (TSG101; ab30871, Abcam), and ALG-2-interacting protein X (Alix; ab186429, Abcam). Thereafter, the membrane was washed three times with TBS-T as described above and then incubated with horseradish peroxidase-conjugated goat anti-rabbit IgG H&L (1:2000 dilution; ab205718, Abcam) for 1 h, at ambient temperature. Finally, after 5 min of signal development, the bands were visualized using an enhanced chemiluminescence detection system (Son et al., 2017).

### Cellular uptake of the extracellular vesicles in vitro

To examine cellular uptake of the EVs in a 2D setup, 5 × 10^5^ cells/mL of MLg (normal murine lung cells), 4T1, and CT26 cells were first, respectively, cultured overnight in confocal dishes in an incubator. Thereafter, the cells were treated with CT26-EV-DOX (at a DOX concentration of 10 µg/mL) for 12 h. After incubation, the cells were washed three times with PBS, fixed with 4% paraformaldehyde (PFA), counterstained with 4′,6-diamidino-2-phenylindole (DAPI; Thermo Fisher Scientific, Waltham, MA, USA), and visualized using a confocal microscope.

Fluorescence-activated cell sorting (FACS) by flow cytometry was used to quantify the cellular uptake of EVs. The various cell lines (5 × 10^5^ cells/mL) were cultured in 6-well plates and treated as described above. After 12 h, the cells were washed three times with PBS, fixed with 4% PFA, and analyzed with a FACS system (MACS, Miltenyi Biotec, Auburn, CA, USA).

To examine cellular uptake by 3D tumor spheroids, an *in vitro* 3D model of CT26 tumor cells was first induced in ultra-low attachment plates (Corning, Corning, NY, USA). In brief, 2 × 10^3^ CT26 cells were added to each well of the plate containing 100 μL of Dulbecco’s modified Eagle’s medium (DMEM). After centrifugation at 1200 rpm for 3 min, the cells were left in an incubator for 3 days. Once spheroids had formed, the medium in the well was replaced with 100 µL of DMEM containing DOX, 4T1-EV-DOX, or CT26-EV-DOX (all at a DOX concentration of 10 µg/mL), and the plates were further incubated for 12 h. Then, after washing the spheroids three times with PBS, they were transferred to confocal dishes, fixed with 4% PFA, rinsed with PBS, stained with DAPI, and finally observed using the confocal microscopy system.

### In vitro cytotoxicity test

The 3-(4,5-dimethylthiazol-2-yl)-2,5-diphenyltetrazolium bromide (MTT) assay was used to evaluate the toxicity of drug-free CT26-EV nanoparticles at different EV concentrations (0–500 µg/mL) toward MLg and CT26 cells. Additionally, MLg, 4T1, and CT26 cells were, respectively, treated with CT26-EV-DOX nanoparticles of different DOX concentrations (0–500 ng/mL) for 24 h. After the treatment, the cells were rinsed three times with PBS, and then 10 µL of MTT (Sigma-Aldrich, St. Louis, MO, USA) in 100 µL of DMEM was added to each well. After 3.5 h, the culture media were discarded, and an equal volume of dimethyl sulfoxide solution was added. The absorbance of the contents in each well was measured at 570 nm using a multimode microplate reader.

### Animal model

We used BALB/c mice (Orient Bio Inc., Seoul, Korea) as the animal model for this study (Nguyen et al., 2019). All animal study was performed with the permission of the Ethics Committee of Chonnam National University under the license numbers (CNU IACUC-YB-2021-77), in compliance with protocols approved by the Institutional Animal Care and Use Committee (IACUC) of Chonnam National University. Colorectal tumors were induced in the mice by subcutaneously injecting 100 μL of 1 × 10^6^ CT26 cells (in PBS) into the right flank of each mouse. The tumors formed approximately 10 days after the injection, reaching an average size of 100 mm^3^.

### Tumor targeting by the extracellular vesicles and their biodistribution in vivo

The tumor-bearing mice were anesthetized and then intravenously injected through the tail vein with either 4T1-EV or CT26-EV, which had been previously labeled with a 0.25 mg/mL solution of 1,1′-dioctadecyl-3,3,3′,3′-tetramethylindodicarbocyanine, 4-chlorobenzenesulfonate salt (DiD; Thermo Fisher Scientific) according to the manufacturer’s instruction. For the control group, tumor-bearing mice were injected with PBS only. At 1, 3, 5, and 24 h post injection, DiD signals from EVs were detected at the excitation/emission wavelengths of 644/665 nm using an *in vivo* imaging system (LB 983, NightOWL II, Berthold Technologies, Bad Wildbad, Germany) (Pang et al., 2018). Next, to observe the *ex vivo* biodistribution of the signals in major organs of the mice, the animals were sacrificed and the organs were excised and imaged using the same *in vivo* imaging system at the same wavelengths. Finally, the mean fluorescence intensity from each organ was evaluated quantitatively using the indiGO software application built into the LB 983 system.

### Therapeutic study in vivo

CT26 tumors were induced in BALB/c mice using the method described above. Upon tumor formation, the animals were randomly divided into five groups (*n* = 5 per group), anesthetized, and intravenously injected with one dose of PBS, CT26-EV, DOX, 4T1-EV-DOX, or CT26-EV-DOX (all at a DOX concentration of 1 mg/mL) every 3 days, for a total of five doses from days 0 to 12. Tumor growth in the animals was observed for 14 days. The tumor size was calculated using the following formula: *L* × *W*^2^/2, where *L* and *W* are the longest and shortest dimensions of the tumor, respectively (Wang et al., 2013; Nguyen et al., 2019).

### Blood biochemistry

Mice were treated with the different samples as described above. On day 7 after injection, the animals were euthanized and blood (1 mL per mouse) was collected via cardiac puncture. The whole-blood samples were centrifuged at 2000 ×*g* for 10 min at 4 °C, and the sera were collected for determination of their levels of the liver function indicators alanine transaminase (ALT), aspartate transaminase (AST), and alkaline phosphatase (ALP) and the kidney function indicators blood urea nitrogen (BUN) and creatinine (Cre), using an automated dry chemistry analyzer (DRI-CHEM 700i, Fujifilm, Tokyo, Japan) (Nguyen et al., 2021).

### Hematoxylin and eosin examination

For histological examination of the organ tissues, the mice were sacrificed at day 7 post injection, and the major organs (i.e. heart, spleen, liver, kidney, and lung) and tumors were dissected, fixed in 4% PFA, and embedded in paraffin before further use. Tissue sections of 5 μm thickness were obtained using a microtome and mounted onto adhesive glass slides. The sectioned tissues were then stained with hematoxylin and eosin (H&E) according to the standard protocol and analyzed under an optical microscope (Huang et al., 2017).

### Statistical analysis

Data are presented as the means ± standard deviation of three or more samples. One-way analysis of variance followed by Tukey’s multiple comparison test was performed to determine the statistical significance of differences between pairs of groups using GraphPad Prism (GraphPad Software, Inc., San Diego, CA, USA). Additionally, pairwise comparisons were performed using the Student *t*-test. A *P* value of less than 0.05 was considered statistically significant.

## Results

### Preparation of CT26-EV-DOX nanoparticles

**Figure 2.**
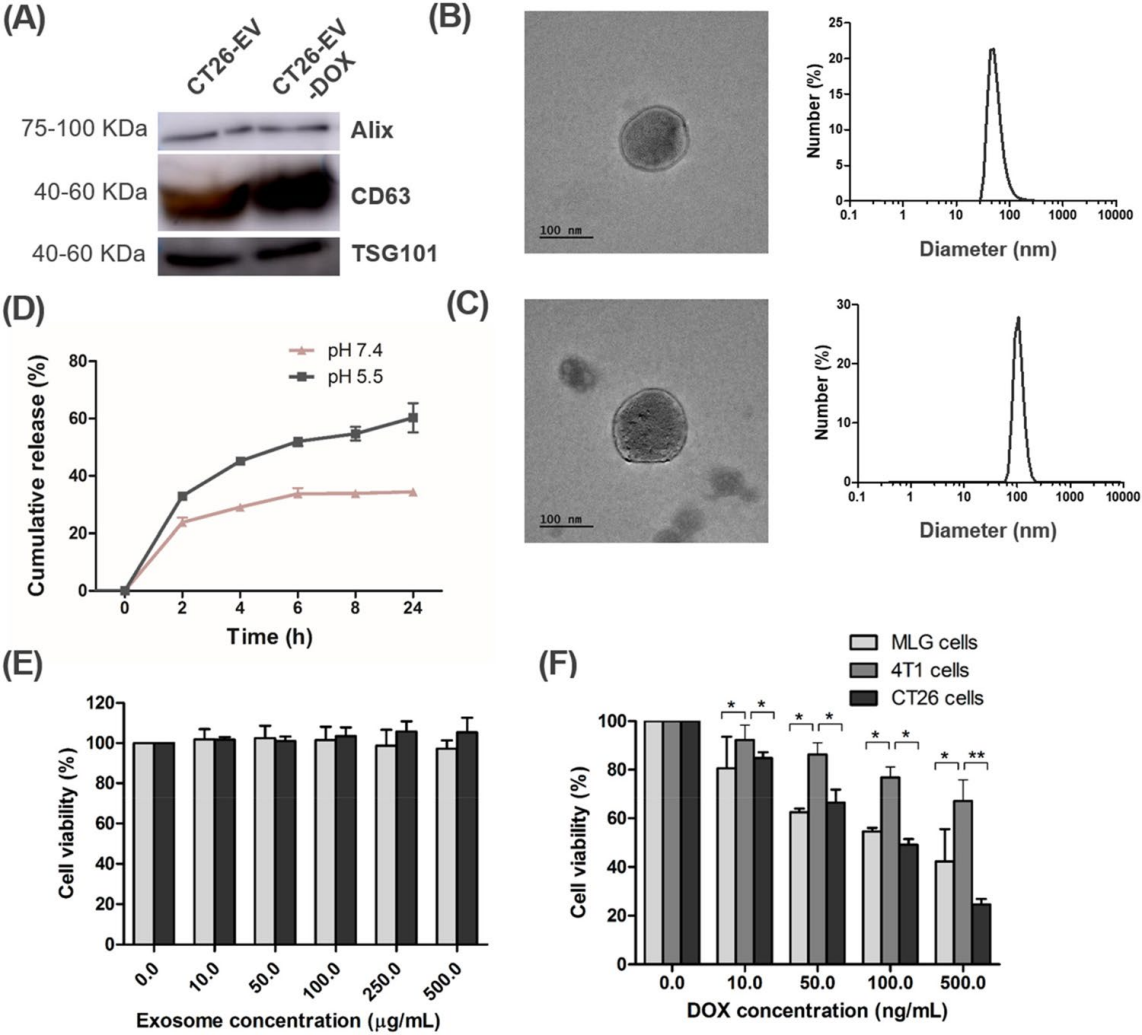
Characterization of the CT26-DOX-EV nanoparticles. (A) Western blot images of EV markers before and after DOX loading. (B, C) TEM images and size distribution of CT26-EV (B) and CT26-EV-DOX (C). (D) Drug release patterns of CT26-EV-DOX at pH 5.5 and 7.4. (E, F) Viability of MLg, 4T1, and CT26 cells treated with different concentrations of CT26-EV (E) and CT26-EV-DOX (F) for 24 h. The bars represent the SD (*n* = 3; *P < .05, **P < .01). DOX, doxorubicin; EV, extracellular vesicles; MLg, normal murine lung cells; 4T1, murine mammary carcinoma cells; CT26, murine colorectal cancer cells.

EVs were prepared that had been extracted from CT26 and 4T1 tumor cells (cultured in medium containing EV-depleted fetal bovine serum) using serial centrifugation steps. Western blot assays showed that the lysate in the centrifugation pellet contained the EV markers Alix, CD63, and TSG101 (Figure 2A), thus confirming that it contained EVs (Wang et al., 2018; Zhang et al., 2018; Kim et al., 2019). The anticancer drug DOX was then loaded into the CT26-EV nanoparticles via electroporation (Gune Pulser Xcell, Bio-Rad, Hercules, CA, USA). DOX concentration was determined by measuring fluorescent intensity of the EV lysates based on the constructed standard curve of known concentration of DOX. Free DOX was removed by ultracentrifugation. By varying different electroporation parameters and DOX feeding amounts for 100 µg of EVs, we found the optimal drug-loading conditions to be an electroporation voltage of 350 V, a capacitance of 150 µF, and a DOX amount of 50 µg, which resulted in 26.35 and 26.21 µg of DOX/100 µg of CT26-EV and 4T1-EV, respectively. This loading efficiency was equivalent to that reported in the literature (Yong et al., 2019). The CT26-EV-DOX nanoparticles still retained all three EV markers, suggesting that drug loading had not affected the properties of the EVs (Figure 2A). As evident upon TEM imaging, the EVs had a spherical surface before and after drug loading. Dynamic light scattering experiments revealed that both CT26-EV and CT26-EV-DOX had a narrow size distribution range, with average diameters of 164.4 and 217.9 nm, respectively (Figure 2B and 2C). In addition, Figure S1 shows low magnification TEM image of CT26-EV-DOX. The particle size in these ranges may facilitate uptake of the EVs by the cancer cells via enhanced permeability and retention (EPR) effects.

### In vitro drug release study

The drug release behavior of CT26-EV-DOX over a 24 h period was investigated at two pH values: 7.4 (resembling the blood environment) and 5.5 (resembling the endosome environment). Under both pH conditions, DOX was released from the EVs in a controlled manner without any release bursts. However, a significantly higher amount of the drug was released at pH 5.5 (60.28 ± 5.09%) than at pH 7.4 (32.42 ± 0.7%) after 24 h (Figure 2D). These results indicate that when CT26-EV-DOX particles are circulating in the bloodstream, premature drug release in the blood environment would be minor, thereby minimizing any side effects of the drug.

### Cytotoxicity of the various extracellular vesicles

First, we tested the biocompatibility of DOX-free CT26-EV in the MLg (normal cells) and CT26 cell lines using the MTT assay. After 24 h, CT26-EV did not show any toxicity toward the treated cells, even at concentrations up to 500 µg/mL. The results confirmed the excellent biocompatibility of the prepared EVs (Figure 2E).

Next, we verified the toxicity of CT26-DOX-EV toward normal (MLg) and cancerous cells (4T1 and CT26) at different DOX concentrations (0–500 ng/mL) for 24 h. The toxicity of CT26-EV-DOX toward all tested cell lines was dose dependent, with increasing DOX concentrations resulting in increased cell death. Additionally, at all DOX concentrations, the CT26 (EV parent) cells were the most susceptible to the cytotoxic effect of CT26-EV-DOX, whereas the 4T1 cells were the least susceptible (Figure 2F). These results indicated that the prepared CT26-EV-DOX nanoparticles specifically targeted CT26 cells rather than cells of other tumor types.

### In vitro cellular uptake in a 2D setup

**Figure 3.**
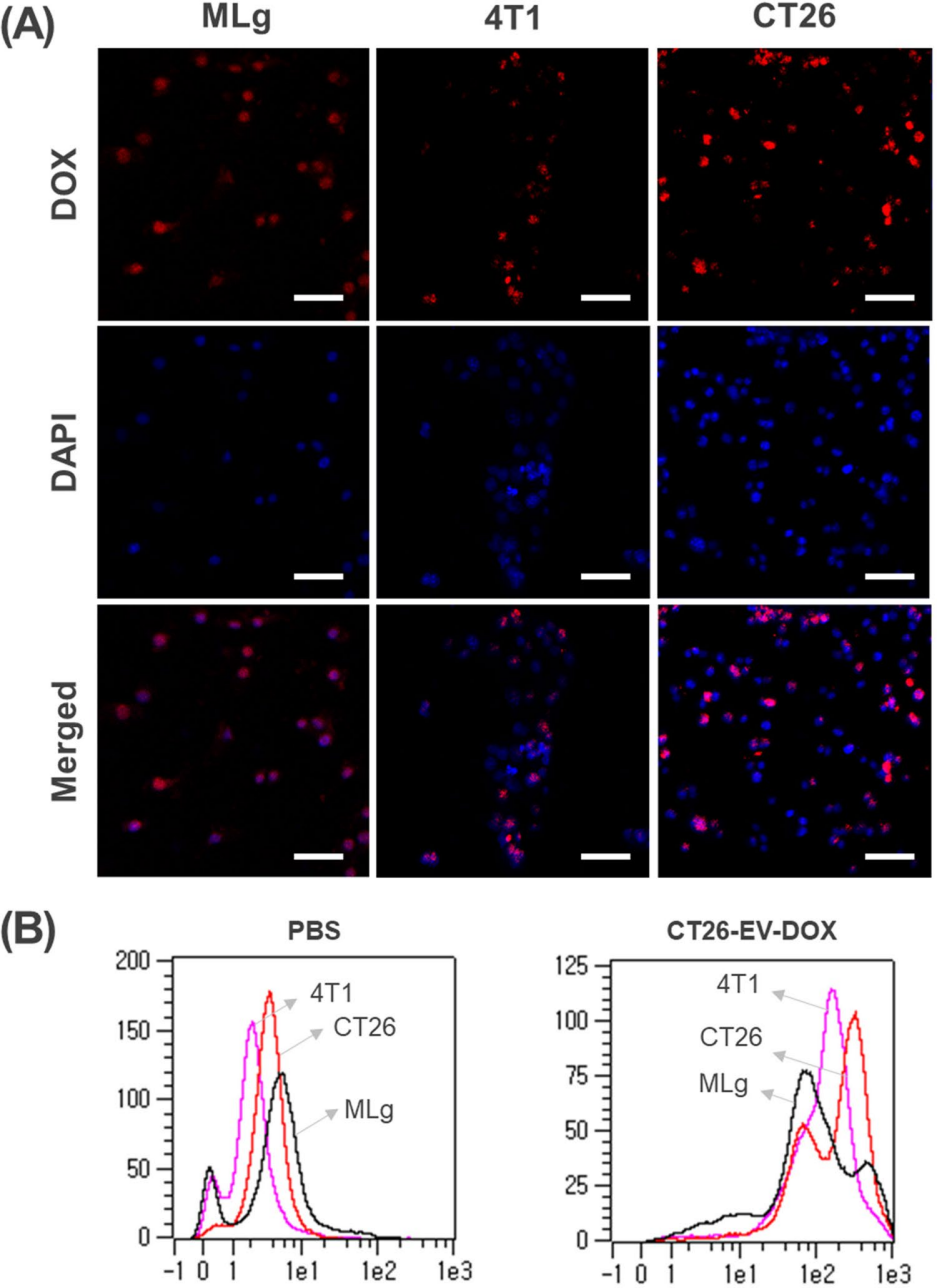
(A) Confocal laser scanning microscopy images showing the cellular uptake of CT26-EV-DOX by MLg, 4T1, and CT26 cells, scale bar = 50 µm. (B) FACS quantification of the signals in (A). DOX, doxorubicin; EV, extracellular vesicles; MLg, normal murine lung cells; 4T1, murine mammary carcinoma cells; CT26, murine colorectal cancer cells; PBS, phosphate-buffered saline.

The cellular uptake of CT26-EV-DOX was first assessed using confocal laser scanning microscopy (CLSM). Owing to its intrinsic fluorescence property, DOX appears red under CLSM at the excitation/emission wavelengths of 488/564–606 nm. DAPI was used for nuclear staining. As shown in Figure 3(A), a higher density of red signals was observed in the CT26 cells than in the MLg and 4T1 cells, confirming that the CT26-EV-DOX displayed higher affinity toward its parent cells. The confocal images of the cells treated with control sample (PBS) were presented in Figure S2. In addition, for reference we also prepared the confocal images of cellular uptakes 4T1-EV-DOX by the cells, which were displayed in Figure S3. Altogether, the targeting of tumor cells with EVs derived from the same type of tumor would reap higher benefits than that using EVs derived from cells of a different tumor type. The results may be explained by the role of adhesion molecules on the surface of the CT26-EV-DOX nanoparticle, which may give it a higher ability to target the parent cells. Moreover, the EVs could display receptors that bind with specific ligands on the tumor cell membrane, as suggested recently by Qiao et al. (2020).

Additionally, the cellular uptake of CT26-EV-DOX was evaluated quantitatively using flow cytometry (FACS). In agreement with the CLSM results, the CT26 cells took up a significantly higher amount of the CT26-EV-DOX nanoparticles than the MLg and 4T1 cells did (Figure 3B).

### In vitro cellular uptake by 3D tumor spheroids

**Figure 4.**
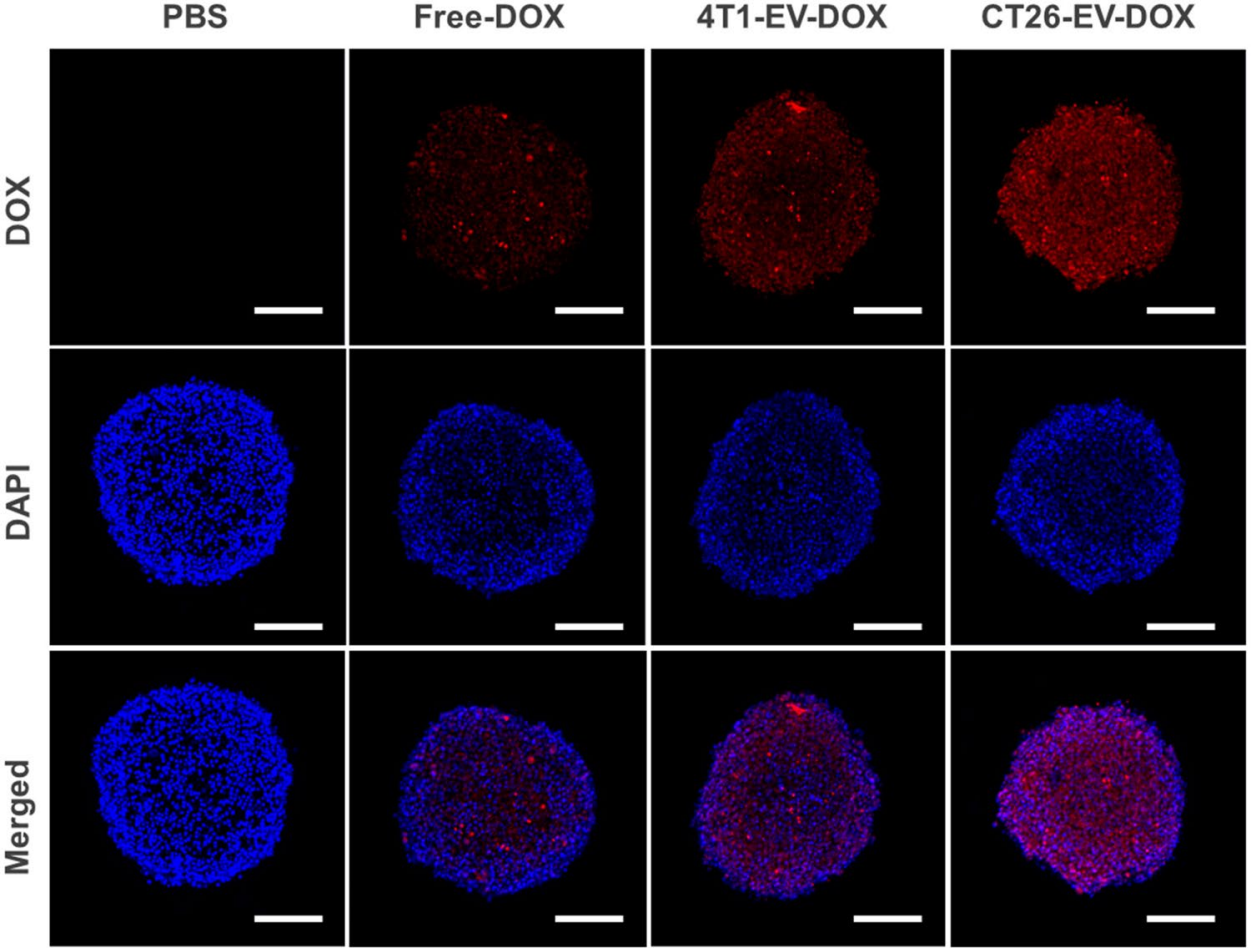
Cellular uptake of the free drug or drug-loaded extracellular vesicles by 3D tumor spheroids. DOX, doxorubicin; EV, extracellular vesicles; PBS, phosphate-buffered saline, scale bar = 200 µm.

Colorectal cancer is a type of solid tumor. Therefore, to precisely model the targeting of the EVs to and their uptake by the tumors in a way that resembles the *in vivo* condition, 3D tumor spheroids should be used. In this study, we induced the formation of CT26 tumor spheroids using ultra-low attachment cell culture plates and then treated them with different samples (i.e. PBS, free DOX solution, 4T1-EV-DOX, and CT26-EV-DOX, with an equivalent DOX concentration of 10 µg/mL) for 12 h in the incubator. As indicated in Figure 4, the highest DOX signal was observed in the group treated with the CT26-EV-DOX nanoparticles, thus confirming their excellent abilities in targeting and penetrating their parent cancer cells. To confirm that the extracellular vesicles were not merely attached to the surfaces of the spheroids, we performed the imaging at different cut planes using the Z-stack function of the CLSM system. Accordingly, we could observe the red signal of DOX at different focal depths, thereby verifying that the EVs had the ability to penetrate deeply within the tumor spheroids (Figure S4).

### In vivo tumor targeting by the extracellular vesicles and their biodistribution

**Figure 5.**
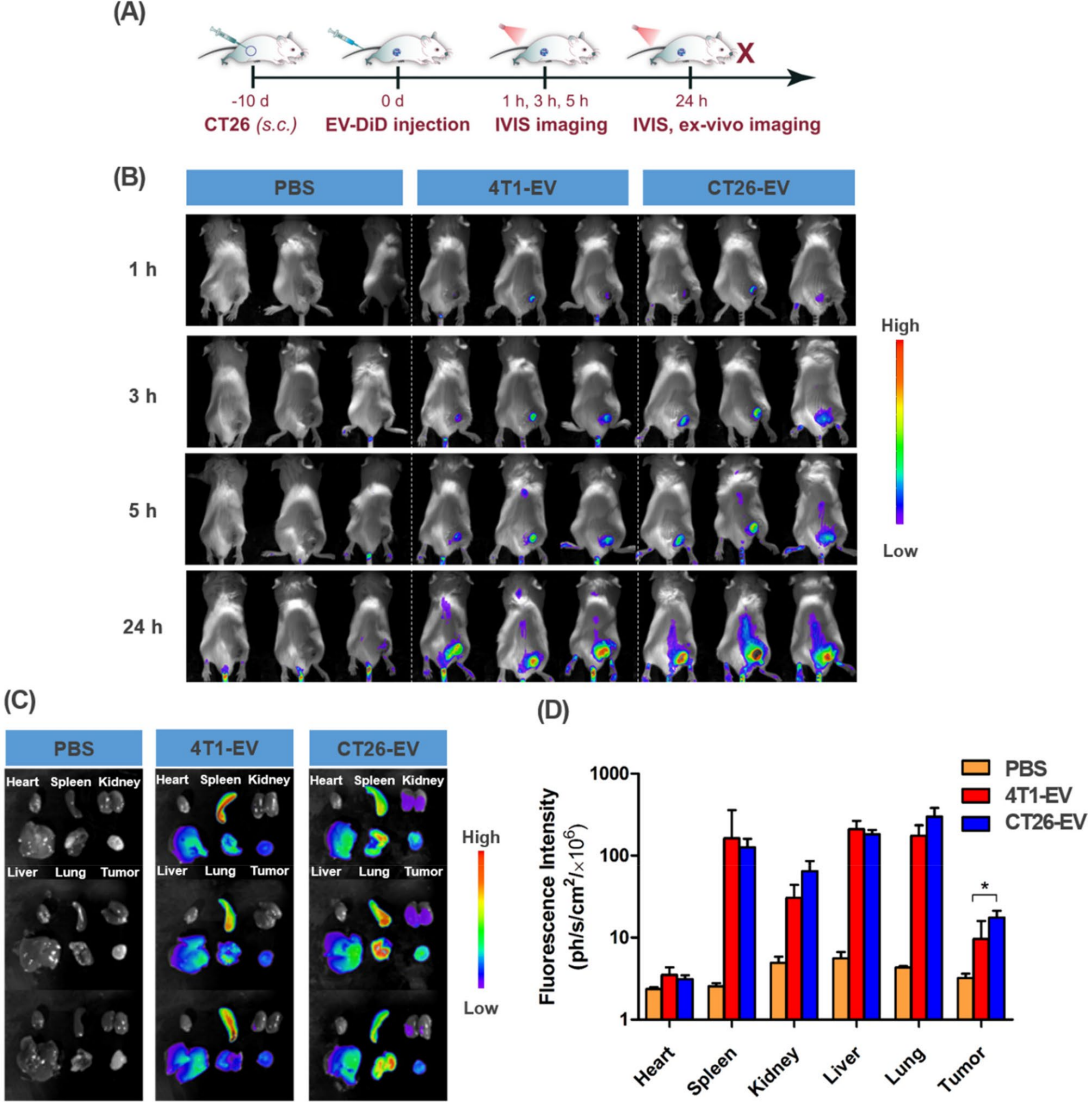
*In vivo* assay of the tumor-targeting abilities of extracellular vesicles in CT26 tumor-bearing BALB/c mice. (A) Schematic description of the experiment. (B) IVIS images of the mice at different time points. (C) *Ex vivo* fluorescence images of major organs of the mice in different treatment groups at 24 h post injection. (D) Quantification of the fluorescence signals in (A). The bars represent the SD (*n* = 3; *P < .05). IVIS, *in vivo* imaging system; DiD, 1,1′-dioctadecyl-3,3,3′,3′-tetramethylindodicarbocyanine, 4-chlorobenzenesulfonate salt; DOX, doxorubicin; EV, extracellular vesicles; 4T1, murine mammary carcinoma cells; CT26, murine colorectal cancer cells; PBS, phosphate-buffered saline.

To assess the tumor-targeting ability of the EVs *in vivo*, we labeled them with DiD. CT26 tumor formation was induced in 6–8-week-old BALB/c mice by inoculating 1 × 10^6^ cells into the right flank of the animals. After 10 days, when the tumor volume was approximately 100 mm^3^, the mice were intravenously injected with PBS, DiD-labeled 4T1-EV, or DiD-labeled CT26-EV. Fluorescence images were taken at 1, 3, 5, and 24 h post injection. As shown in Figure 5(A), the DiD signal gradually increased with time in the groups treated with 4T1-EV and CT26-EV, thus confirming the tumor-targeting abilities of these two types of EVs. However, the fluorescence signals were significantly higher in the group injected with CT26-EV, thereby verifying their excellent ability in homing in on their parent cancer cells (Figure 5B). After 24 h, the mice were sacrificed, and the main organs were harvested and imaged using the same *in vivo* imaging system. As shown in Figure 5(C), DiD signals were detected in all major organs of the mice treated with EVs. However, the signals from the tumors of the CT26-EV-treated mice were significantly higher than those from the tumors of the 4T1-EV-treated mice. Quantitative analysis of the fluorescence intensity further confirmed these results (Figure 5D).

### Therapeutic effects of the drug-loaded extracellular vesicles in vivo

**Figure 6.**
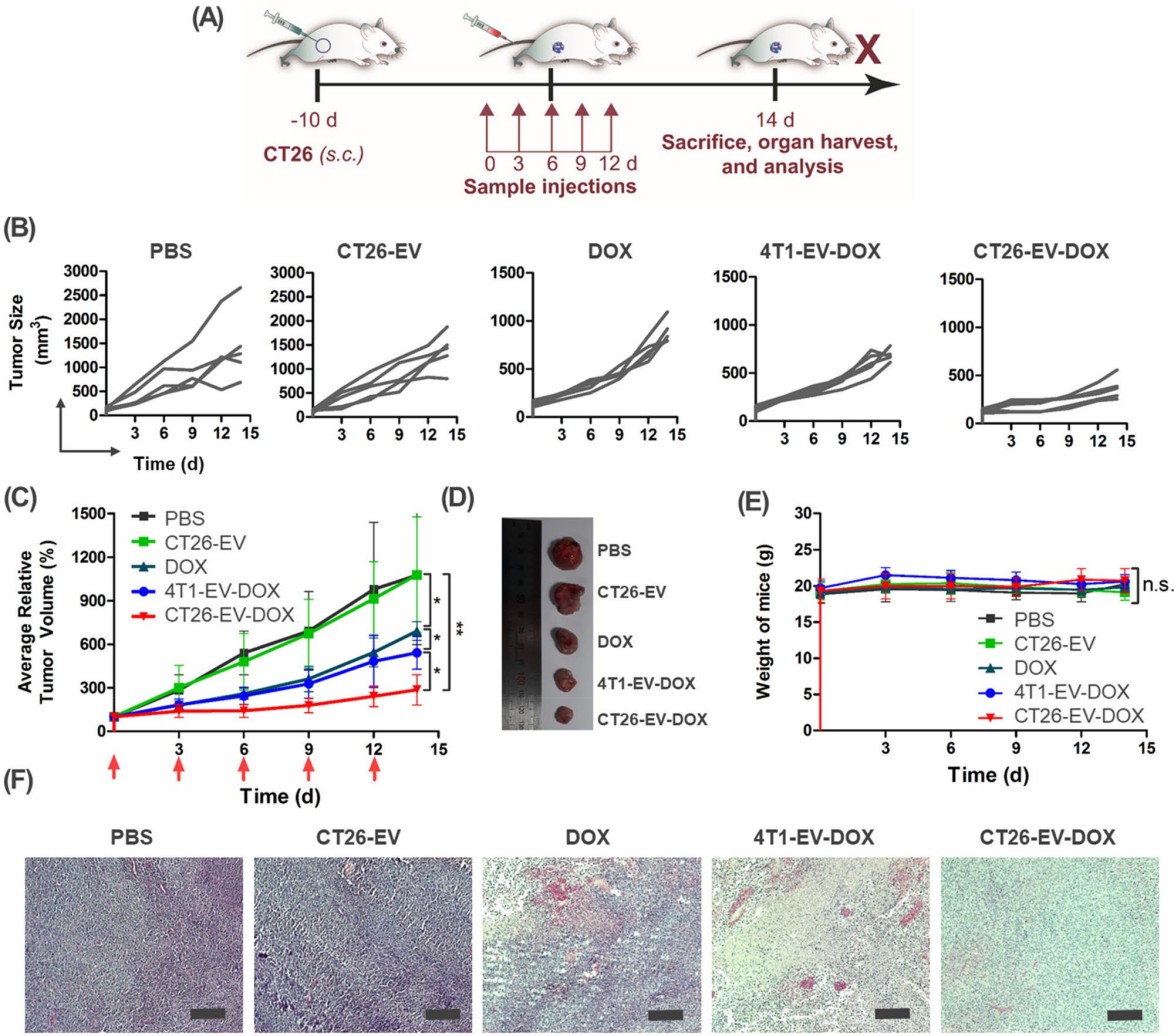
*In vivo* therapy experiment. (A) Schematic diagram of the experiment. (B) Tumor growth of the individual mouse in each group. (C) Comparison of tumor growth among all treatment groups. (D) Representative images of the tumor mass dissected from mice in each group. (E) Weight loss of the mice during the treatment course. (F) Representative images of H&E-stained tissue sections of the tumor mass harvested from mice in all treatment groups, scale bar = 200 µm. In (C) and (E), the bars represent the SD (*n* = 3; *P < .05, **P < .01). DOX, doxorubicin; EV, extracellular vesicles; 4T1, murine mammary carcinoma cells; CT26, murine colorectal cancer cells; PBS, phosphate-buffered saline.

Next, we verified the therapeutic effects of the drug-loaded EVs in CT26 tumor-bearing BALB/c mice. Once the tumor volume had reached approximately 100 mm^3^ (after 10 days), the mice were intravenously injected with one dose of PBS, CT26-EV, free DOX, 4T1-EV-DOX, or CT26-EV-DOX (all with 5 mg/kg of DOX) every 3 days for a total of five doses (Figure 6A). Figure 6(B) shows the size of the tumor from each mouse in each group. The comparison of the average relative tumor volumes of all treatment groups is shown graphically in Figure 6(C). It was obvious that PBS and drug-free EVs had no therapeutic effect against the tumors, whereas the free DOX solution induced some delay in tumor growth. Importantly, although both the CT26-EV-DOX and 4T1-EV-DOX groups showed critically delayed rates of tumor growth, the tumor-inhibiting effect of CT26-EV-DOX was far more superior (Figure 6D). These results further confirm the therapeutic excellence of CT26-EV-DOX against colorectal tumors. In addition, none of the mice in any of the groups exhibited critical weight loss during the treatment course (Figure 6E). Moreover, histological H&E staining of tumor slices from the various mouse groups revealed the absence of apoptotic cells in the animals treated with PBS and CT26-EV. By contrast, critical tissue damage and apoptotic cells were obvious in the mice treated with DOX and 4T1-EV-DOX and especially so in those treated with CT26-EV-DOX (Figure 6F).

Additionally, we evaluated the mouse serum levels of liver (ALP, ALT, and AST) and kidney function indicators (Cre and BUN). All liver and kidney indicators in the five mouse groups remained within the normal ranges, thus further confirming the excellent *in vivo* biocompatibility of the prepared EVs (Table S3). These results were verified through histological (H&E) analysis of the major organ tissues, which also highlighted the absence of toxicity in the examined specimens (Figure S5). In particular, no cardiotoxicity was observed in all groups, including the free DOX group. The possible reason was that cumulatively low doses of the drug (5 mg/kg) were used. It was reported by Desai et al. that the severity of cardiac lesions was observed in mice injected with a DOX cumulative dose of 24 mg/kg or higher (Desai et al., 2013). The results suggested that using the developed formulation, the amounts of administered drugs could be reduced while maintaining a great therapeutic efficacy.

## Discussion

It is widely known that using nanoparticles to deliver therapeutic drugs to tumor would increase the overall therapeutic efficiency of treatment of the tumor (Wilhelm et al., 2016). Since the sizes of the particles are nano-scaled that would facilitate the penetration into the tumor via EPR effect. As an emerging platform for nanoparticle-based drug delivery, extracellular vesicles are biocompatible, biodegradable (Qiu et al., 2019). And especially they share identical lipid composition and protein with those of their parent’s cells, thus they may preferentially interact with the cells. Recently, Qiao and coworkers found that EV integrins may drive the tumor-specific colonization of tumor derived extracellular vesicles using proteome assay analysis (Qiao et al., 2020). For the above reasons, using tumor derived extracellular vesicles would provide an innovative means of targeting and delivering therapeutic drugs to the extracellular vesicle’s parent cells. Regarding tumor-derived EV formulation developments, recently Yong et al. isolated extracellular vesicles by incubating DOX loaded porous silicon nanoparticles to tumor cells and demonstrated that the nanoparticles are efficient drug delivery systems for chemotherapy of cancer (Yong et al., 2019). Qiu et al. developed EV nanoparticles from breast cancer cells and showed that the nanovesicles promote lung distribution of the therapeutics via repression of Kupffer cell-medicated phagocytosis (Qiu et al., 2019). Qiao et al. isolated extracellular vesicles from a fibrosarcoma cell line (HT1080) and a cervical cancer cell line (HeLa) loaded with DOX contained liposomes (Doxil) and revealed that the extracellular vesicles exert excellent targeting to their original cell lines with promising *in vivo* therapeutic effect (Qiao et al., 2020). In the present work, we proposed a unique formulation specific targeting to colorectal tumor using extracellular vesicles derived from the same tumor cell line. In addition, we employed simple and effective DOX loading process into the extracellular vesicles using electroporation, which resulted in high drug loading capacity. The average size of the extracellular vesicles was in the range of 200 nm, which also facilitated cellular uptake via EPR effect (Nakamura et al., 2016). In addition, when compared with extracellular vesicles isolated from 4T1 cancer cells (4T1-EV-DOX), the developed CT26-EV-DOX displayed excellent cellular uptake to CT26 cells both in 2D and 3D cell settings. The extracellular vesicles also showed good tumor homing ability and exerted promising therapeutic outcomes *in vivo*. The enhanced cellular uptake and homing of CT26-EV-DOX in comparison with those of their counterparts may be attributed to the shared lipid compositions and proteins with hemophilic adhesion domains, namely focal adhesion proteins, integrin, and family of RHO proteins (Qiao et al., 2020) of the extracellular vesicles and their parent cells. Based on these results, our future works will focus on understanding the underlying mechanism of the interaction of the extracellular vesicles with their cells of origin. In addition, to make the results of the works closer to clinical application, we will isolate extracellular vesicles from real cancer patients and loaded with therapeutic drug. In order to further enhance the tumor targeting, tumor specific ligands will be grafted on the extracellular vesicles surface. In combination with the use of magnetic nanoparticles, the extracellular vesicles will be better driven to the targeted area with the help of electromagnetic actuating system or clinical magnetic resonance imaging systems (Felfoul et al., 2016; Go et al., 2021).

## Conclusion

In summary, we isolated EVs from colorectal cancer cells and engineered them for homing to their parent cells after they had been loaded with DOX. The *in vitro* and *in vivo* experiments on the performance of the CT26-EV-DOX nanoparticles revealed their excellent tumor-targeting ability and therapeutic effect against CT26 tumors. These results suggest that these novel CT26-EV-DOX nanoparticles could be an effective alternative nanomedicine for treating patients with colorectal cancer.

## Supplementary Material

Supplemental Material

## Data Availability

The data presented in this study are available on request from the corresponding author.
