# Supplementary material for: Tumor-derived extracellular vesicles for the active targeting and effective treatment of colorectal tumors in vivo
Source: Drug Deliv. 2022 Aug 8;29(1):2621–31. doi: 10.1080/10717544.2022.2105444 (PMC9367655; doi:10.1080/10717544.2022.2105444)
Supplement: Supplemental Material [file IDRD_A_2105444_SM2660.docx]

Supplementary Materials

Tumor-derived Extracellular Vesicles for the Active Targeting and Effective Treatment of Colorectal Tumors *in Vivo*

Van Du Nguyen^1,2^, Ho Yong Kim^2^, You Hee Choi^2^, Jong-Oh Park^2*^, Eunpyo Choi^1, 2,*^

1. School of Mechanical Engineering, Chonnam National University, 77 Yongbong-ro, Buk-gu, Gwangju 61186, Korea
2. Korea Institute of Medical Microrobotics, 43-26, Cheomdangwagi-ro 208-Beon-gil,
   Buk-gu, Gwangju 61011, Korea.

**Table S1**. Comparative studies of drug delivery using extracellular vesicles

| **No.** | **Parent cell** | **Isolation method** | **Size**  **(d, nm)** | **Drug/Loading method** | **DOX loading** | **Targeted disease** | **In vitro 3D targeting** | **In vivo targeting/ therapy** | **Reference** |
| --- | --- | --- | --- | --- | --- | --- | --- | --- | --- |
| **1** | **CT26** | **Ultra-centrifugation** | **217.9** | **DOX/**  **Electroporation** | 26.35 µg/100µg^-1^ protein | **Colorectal cancer** | **Yes** | **Yes** | **This Work** |
| 2 | THP-1 | Ultra-centrifugation | 141.2 | DOX/ Electroporation | N/A | Breast cancer | No | Yes | Biomaterials (2021) [[1](#_ENREF_1)] |
| 3 | RAW264.7 | Ultra-centrifugation | 150 | aCD47, aSIRPα | 1.1 µg/100µg^-1^ protein | Breast cancer | No | Yes | Angew.Chem. Int. Ed. (2020) [[2](#_ENREF_2)] |
| 4 | Fibroblasts | Centrifugation & Extrusion | 137.5 | DTX/ Membrane fusion | 4.3% | Metastatic Peritoneal Cancer | No | Yes | Adv. Sci. (2020) [[3](#_ENREF_3)] |
| 5 | J774A.1 | Centrifugation & Extrusion | 177 | DOX/Membrane fusion | 1.8% | Osteosarcoma, breast cancer | No | No | Acta Biomaterialia (2019) [[4](#_ENREF_4)] |
| 6 | THP-1 | Ultra-centrifugation | 150-300 | DOX/ Electroporation | 79% | Breast cancer | No | Yes | Adv. Funct. Mater. (2018) [[5](#_ENREF_5)] |
| 7 | RAW264.7 | Ultra-centrifugation | 122.7 | Cur/ Electroporation | 4000 µg/1000µg^-1^ protein | Glioma | No | Yes | Biomaterials (2018) [[6](#_ENREF_6)] |
| 8 | HUVECs | Microfluidics | <150 | PTX, DOX /Incubation | N/A | Liver cancer | No | Yes | ACS Appl. Mater. Interfaces (2017) [[7](#_ENREF_7)] |
| 9 | THP-1 | Magnetic separation | 300-1000 | DOX /Electroporation | N/A | Breast cancer | No | Yes | ACS Nano (2017) [[8](#_ENREF_8)] |
| 10 | Blood | Magnetic separation | 40-110 | DOX/ Incubation | 11 µg/105 µg^-1^ protein | Liver cancer | No | Yes | ACS Nano (2016) [[9](#_ENREF_9)] |
|  |  |  |  |  |  |  |  |  |  |
|  |  |  |  |  |  |  |  |  |  |

| **No.** | **Parent cell** | **Isolation method** | **Size**  **(d, nm)** | **Drug/Loading method** | **DOX loading** | **Targeted disease** | **In vitro 3D targeting** | **In vivo targeting/ therapy** | **Reference** |
| --- | --- | --- | --- | --- | --- | --- | --- | --- | --- |
| **1** | **CT26** | **Ultra-centrifugation** | **217.9** | **DOX/**  **Electroporation** | 263.5 ng/µg^-1^ protein | **Colorectal cancer** | **Yes** | **Yes** | **This Work** |
| 2 | MDA-MB-231 | Ultra-centrifugation | 80-200 | DOX/Sonication | 53.18 (%) | Lung metastasis of breast cancer | No | Yes | *J Control Release (2021)[*[*10*](#_ENREF_10)*]* |
| 3 | HCT-116 | Ultra-centrifugation | 110 | 5-FU and miR-21/ Electroporation | 3.1% of 5-FU and 0.5% of miR-21 | Colorectal cancer | No | Yes | J Nanobiotechnol (2020) [[11](#_ENREF_11)] |
| 4 | 4T1 | Isolation Kit | 150 | DCPy | 88.2% | Breast cancer | No | Yes | Angew.Chem. Int.Ed. 2020 [[12](#_ENREF_12)] |
| 5 | HT1080 | Ultra-centrifugation | 87.7 | Doxil/ Extrusion | N/A | Fibrosarcoma | No | Yes | Theranostics (2020) [[13](#_ENREF_13)] |
| 6 | 4T1 | Ultra-centrifugation | 183.5 | N/A | N/A | Lung metastasis of breast cancer | No | Yes | Theranostics (2019) [[14](#_ENREF_14)] |
| 7 | MDA-MB-231 | ExoQuick™ | 100 | Olaparib/ Electroporation | N/A | Breast cancer | No | Yes | Biomaterials (2018) [[15](#_ENREF_15)] |
| 8 | H22 | Centrifugation | 260 | DOX-loaded PSiNPs/ Incubation | 300 ng/µg^-1^ protein | Hepatocarcinoma, Breast cancer | No | Yes | Nat. Commun. (2018) [[16](#_ENREF_16)] |
| 9 | MDA-MB-231 | Ultra-centrifugation | 93 | DOX/ Electroporation | N/A | Breast cancer | No | Yes | Biomaterials (2014) [[17](#_ENREF_17)] |
|  |  |  |  |  |  |  |  |  |  |

**Table S2**. Comparative studies of drug delivery using tumor-derived extracellular vesicles.





**Figure S1**. TEM image of CT26-EV-DOX at low magnification (×60k).

**
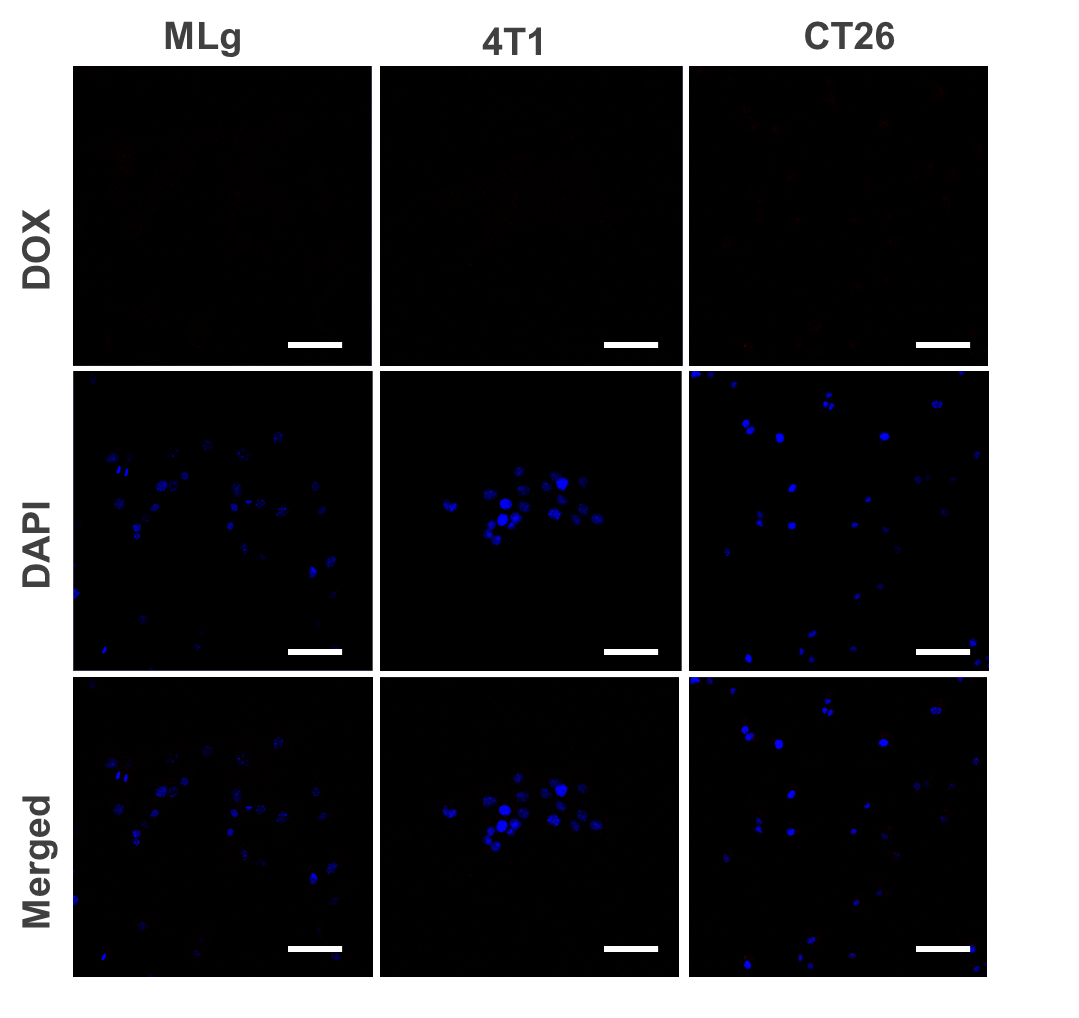
**

**Figure S2**. Confocal laser scanning microscopy images of MLg, 4T1, and CT26 cells treated with phosphate-buffered saline as controls. DOX, doxorubicin; MLg, normal murine lung cells; 4T1, murine mammary carcinoma cells; CT26, murine colorectal cancer cells, scale bar = 50 μm.

**
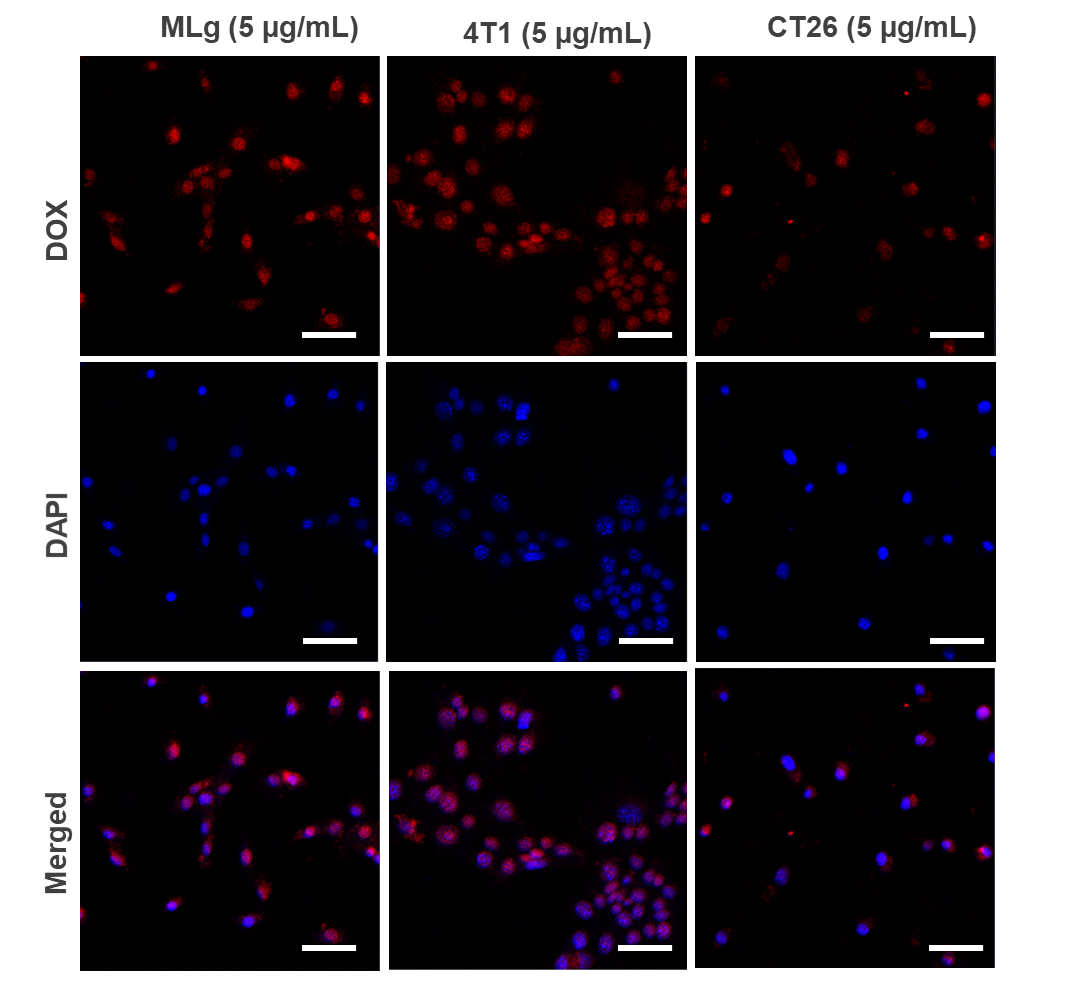
**

**Figure S3**. Cellular uptake of 4T1-EV-DOX by different cell lines. DOX, doxorubicin; MLg, normal murine lung cells; 4T1, murine mammary carcinoma cells; CT26, murine colorectal cancer cells, scale bar = 50 μm.

**
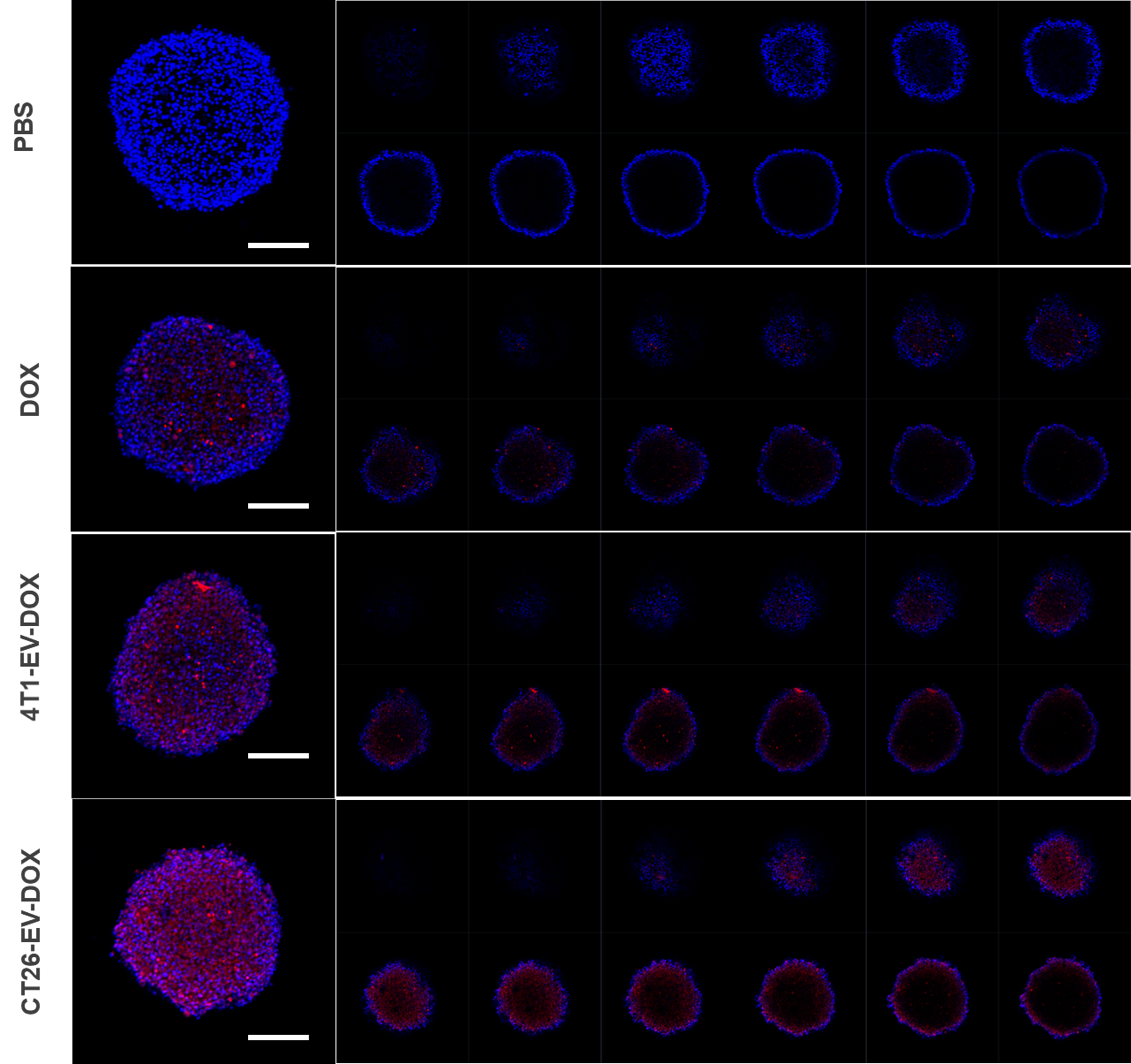
**

**Figure S4**. Confocal laser scanning microscopy images of the different depths of DOX, 4T1-EV-DOX, and CT26-EV-DOX penetration into 3D CT26 tumor spheroids. PBS, phosphate-buffered saline; DOX, doxorubicin; EV, extracellular vesicles; 4T1, murine mammary carcinoma cells; CT26, murine colorectal cancer cells, scale bar = 200 μm.


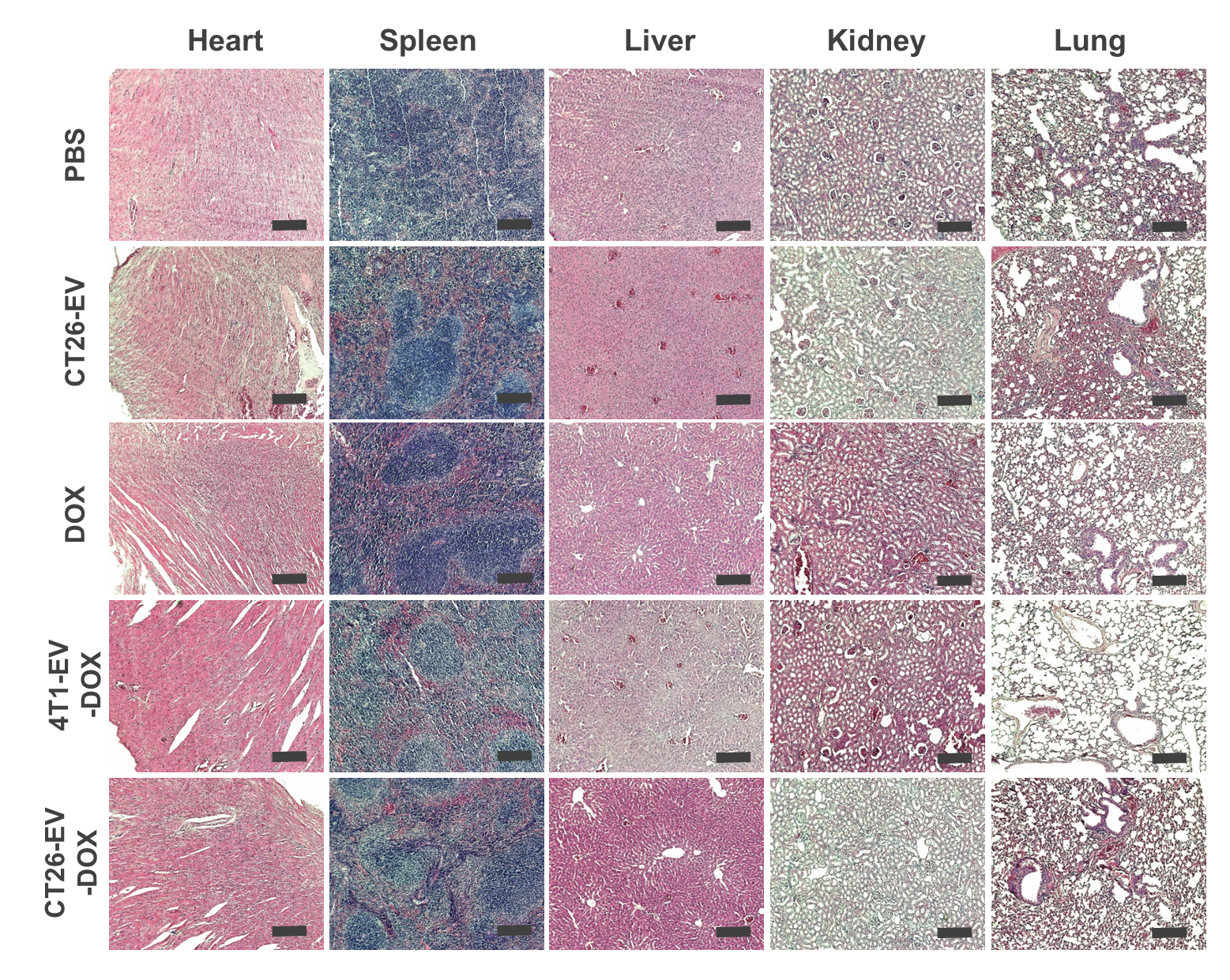


**Figure** **S5**. Representative images of H&E-stained tissue sections of major organs harvested from the mice in all treatment groups. DOX, doxorubicin; EV, extracellular vesicles; 4T1, murine mammary carcinoma cells; CT26, murine colorectal cancer cells; PBS, phosphate-buffered saline, scale bar = 200 μm.

**Table S3**. Blood biochemistry of mice at 14 days after their treatment with different substances.

|  | **ALP**  (U/L) | **ALT**  (U/L) | **AST**  (U/L) | **BUN**  (mg/dL) | **Cre**  (mg/dL) |
| --- | --- | --- | --- | --- | --- |
| **Ref. values*** | No data | 60 | 135 | 18 | 0.4 |
| **PBS** | 170.00 ± 11.00 | 24.67 ± 1.53 | 83.67 ± 7.57 | 18.40 ± 2.25 | 0.14 ± 0.01 |
| **CT26-EV** | 223.33 ± 3.21 | 30.33 ± 3.06 | 100.33 ± 15.01 | 34.13 ± 11.57 | 0.17 ± 0.01 |
| **DOX** | 169.00 ± 12.73 | 24.25 ± 3.54 | 73.00 ± 8.49 | 30.45 ± 14.07 | 0.23 ± 0.09 |
| **4T1-EV-DOX** | 214.33 ± 4.51 | 23.33 ± 2.52 | 73.67 ± 8.02 | 32.63 ± 5.51 | 0.26 ± 0.08 |
| **CT26-EV-DOX** | 205.67 ± 31.66 | 24.33 ± 6.66 | 83.67 ± 17.01 | 24.97 ± 5.74 | 0.20 ± 0.08 |

ALP, alkaline phosphatase; ALT, alanine transaminase; AST, aspartate transaminase; BUN, blood urea nitrogen; Cre, creatinine; PBS, phosphate-buffered saline; DOX, doxorubicin; EV, extracellular vesicles; CT26, murine colorectal cancer cells; 4T1, mammary carcinoma cells.* Ref. values were from Charles River BALB/C mouse biochemistry (aged from 8-10 weeks).

**References**

[1] J. Wang, P. Chen, Y. Dong, H. Xie, Y. Wang, F. Soto, P. Ma, X. Feng, W. Du, B.-F. Liu, Designer exosomes enabling tumor targeted efficient chemo/gene/photothermal therapy, Biomaterials 276 (2021) 121056.

[2] W. Nie, G. Wu, J. Zhang, L.L. Huang, J. Ding, A. Jiang, Y. Zhang, Y. Liu, J. Li, K. Pu, H.Y. Xie, Responsive Exosome Nano-bioconjugates for Synergistic Cancer Therapy, Angew. Chem. Int. Ed. Engl. 59(5) (2020) 2018-2022.

[3] Q. Lv, L. Cheng, Y. Lu, X. Zhang, Y. Wang, J. Deng, J. Zhou, B. Liu, J. Liu, Thermosensitive Exosome–Liposome Hybrid Nanoparticle-Mediated Chemoimmunotherapy for Improved Treatment of Metastatic Peritoneal Cancer, Advanced Science 7(18) (2020) 2000515.

[4] S. Rayamajhi, T.D.T. Nguyen, R. Marasini, S. Aryal, Macrophage-derived exosome-mimetic hybrid vesicles for tumor targeted drug delivery, Acta Biomater. 94 (2019) 482-494.

[5] J. Wang, Y. Dong, Y. Li, W. Li, K. Cheng, Y. Qian, G. Xu, X. Zhang, L. Hu, P. Chen, W. Du, X. Feng, Y.-D. Zhao, Z. Zhang, B.-F. Liu, Designer Exosomes for Active Targeted Chemo-Photothermal Synergistic Tumor Therapy, Adv. Funct. Mater. 28(18) (2018) 1707360.

[6] G. Jia, Y. Han, Y. An, Y. Ding, C. He, X. Wang, Q. Tang, NRP-1 targeted and cargo-loaded exosomes facilitate simultaneous imaging and therapy of glioma in vitro and in vivo, Biomaterials 178 (2018) 302-316.

[7] J. Wang, W. Li, L. Zhang, L. Ban, P. Chen, W. Du, X. Feng, B.-F. Liu, Chemically Edited Exosomes with Dual Ligand Purified by Microfluidic Device for Active Targeted Drug Delivery to Tumor Cells, ACS applied materials & interfaces 9(33) (2017) 27441-27452.

[8] W. Zhang, Z.-L. Yu, M. Wu, J.-G. Ren, H.-F. Xia, G.-L. Sa, J.-Y. Zhu, D.-W. Pang, Y.-F. Zhao, G. Chen, Magnetic and Folate Functionalization Enables Rapid Isolation and Enhanced Tumor-Targeting of Cell-Derived Microvesicles, ACS Nano 11(1) (2017) 277-290.

[9] H. Qi, C. Liu, L. Long, Y. Ren, S. Zhang, X. Chang, X. Qian, H. Jia, J. Zhao, J. Sun, X. Hou, X. Yuan, C. Kang, Blood Exosomes Endowed with Magnetic and Targeting Properties for Cancer Therapy, ACS Nano 10(3) (2016) 3323-3333.

[10] X. Xie, S. Lian, Y. Zhou, B. Li, Y. Lu, I. Yeung, L. Jia, Tumor-derived exosomes can specifically prevent cancer metastatic organotropism, J. Controlled Release 331 (2021) 404-415.

[11] G. Liang, Y. Zhu, D.J. Ali, T. Tian, H. Xu, K. Si, B. Sun, B. Chen, Z. Xiao, Engineered exosomes for targeted co-delivery of miR-21 inhibitor and chemotherapeutics to reverse drug resistance in colon cancer, Journal of Nanobiotechnology 18(1) (2020) 10.

[12] D. Zhu, Y. Duo, M. Suo, Y. Zhao, L. Xia, Z. Zheng, Y. Li, B.Z. Tang, Tumor-Exocytosed Exosome/Aggregation-Induced Emission Luminogen Hybrid Nanovesicles Facilitate Efficient Tumor Penetration and Photodynamic Therapy, Angew. Chem. Int. Ed. Engl. 59(33) (2020) 13836-13843.

[13] L. Qiao, S. Hu, K. Huang, T. Su, Z. Li, A. Vandergriff, J. Cores, P.-U. Dinh, T. Allen, D. Shen, H. Liang, Y. Li, K. Cheng, Tumor cell-derived exosomes home to their cells of origin and can be used as Trojan horses to deliver cancer drugs, Theranostics 10(8) (2020) 3474-3487.

[14] X. Qiu, Z. Li, X. Han, L. Zhen, C. Luo, M. Liu, K. Yu, Y. Ren, Tumor-derived nanovesicles promote lung distribution of the therapeutic nanovector through repression of Kupffer cell-mediated phagocytosis, Theranostics 9(9) (2019) 2618-2636.

[15] K.O. Jung, H. Jo, J.H. Yu, S.S. Gambhir, G. Pratx, Development and MPI tracking of novel hypoxia-targeted theranostic exosomes, Biomaterials 177 (2018) 139-148.

[16] T. Yong, X. Zhang, N. Bie, H. Zhang, X. Zhang, F. Li, A. Hakeem, J. Hu, L. Gan, H.A. Santos, X. Yang, Tumor exosome-based nanoparticles are efficient drug carriers for chemotherapy, Nature communications 10(1) (2019) 3838.

[17] Y. Tian, S. Li, J. Song, T. Ji, M. Zhu, G.J. Anderson, J. Wei, G. Nie, A doxorubicin delivery platform using engineered natural membrane vesicle exosomes for targeted tumor therapy, Biomaterials 35(7) (2014) 2383-2390.
